# There and turn back again: the application of phage serine integrases in eukaryotic systems

**DOI:** 10.3389/fbioe.2025.1478413

**Published:** 2025-02-24

**Authors:** Thais Torquato Sales, Marco Antônio de Oliveira, Lilian Hasegawa Florentino, Rayane Nunes Lima, Elibio Rech

**Affiliations:** ^1^ Embrapa Genetic Resources and Biotechnology, National Institute of Science and Technology in Synthetic Biology, Brasilia, Brazil; ^2^ Department of Cell Biology, Institute of Biological Science, University of Brasilia, Brasilia, Brazil

**Keywords:** serine integrase, eukaryotic cell, phage (bacteriophage), synthetic biology, genome edition tools

## Abstract

Serine integrases (Ints) have gained prominence and have been extensively used in Synthetic Biology due to their ability to modify DNA sequences. Ints are recombinases encoded by the phage genome and have been used to unidirectionally catalyze an insertion, excision, or inversion of a specific DNA sequence between the two attachment sites (*att*) *attB* (bacterial attachment site) and *attP* (phage attachment site). The entire process is highly specific and accurate; therefore, Ints are widely used in genetic engineering and have been extensively studied due to their unique site-specific recombination properties and potential genome editing applications. Furthermore, new recombinational factors (RDFs) and their determinants are constantly being discovered, underlining the need to update progress in research involving Ints in eukaryotic cells. In this way, this review aims to provide an overview of Ints in eukaryotic cells and highlight how Ints can be used in innovative ways to advance genetic engineering applications in health, agriculture, and environmental sciences.

## Graphical Abstract

**Figure:**
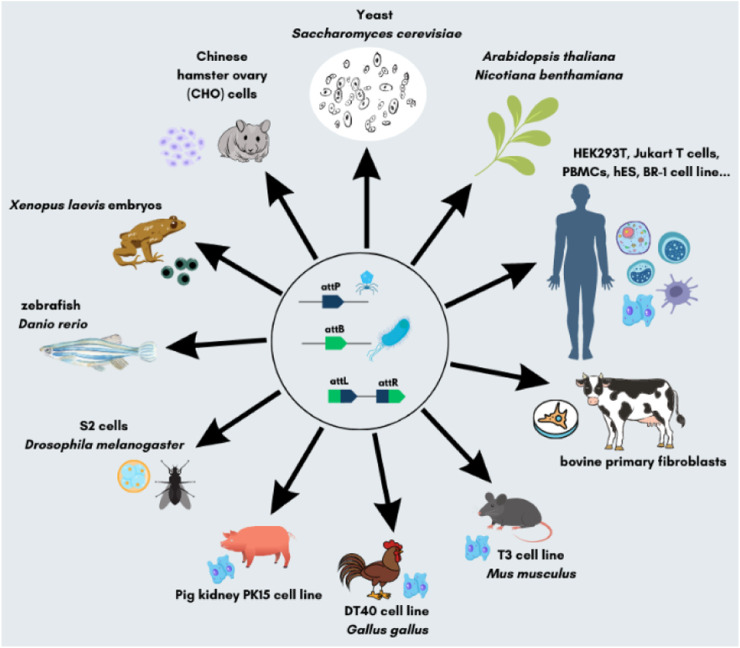
Moving beyond bacteria: unraveling the mechanisms of phage serine integrases in eukaryotic cells to drive biotechnological research, genome editing and therapeutic applications. Created with Canva Pro.

## 1 Introduction

**FIGURE 1:**
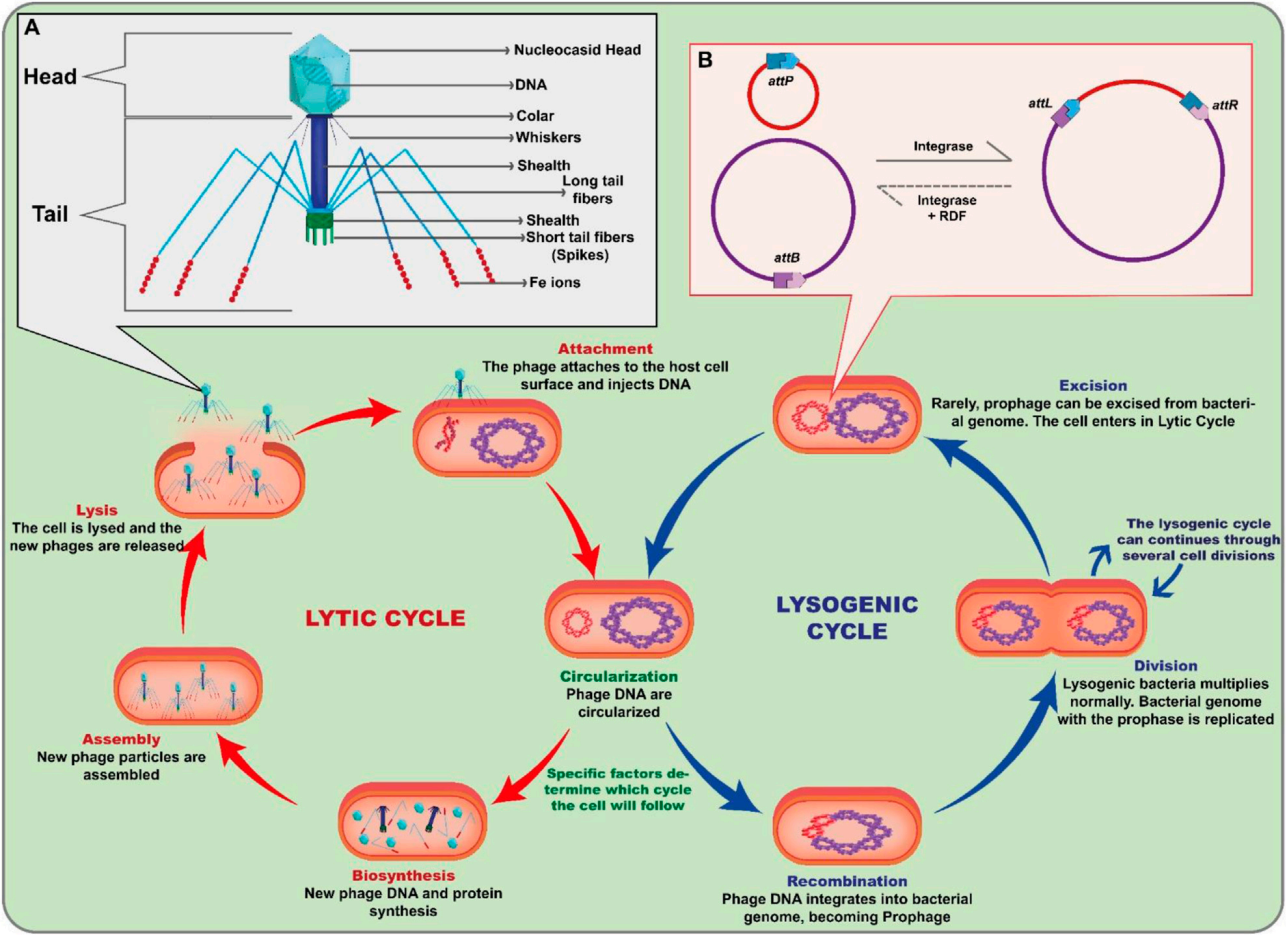
Bacteriophage life cycle and importance of serine-integrases in the process **(A)** The bacteriophage has a structure composed of a protein capsule that surrounds its genetic material. The tail of the phage functions in specific binding and entry of the virus into the host bacterium. It consists of a long tubular structure called a sheath, which connects to the phage head. At the end of the tail, specialized structures such as fibers or fibrils are found, allowing the phage to bind to specific receptors on the surface of the host bacterium. During the lytic cycle, the bacteriophage attaches to the host cell and injects its genetic material, which takes control of the cellular machinery, resulting in the production of new viruses and lysis of the host cell, releasing the newly formed viruses. In the lysogenic cycle, instead of immediately initiating the lytic process, the genetic material of the bacteriophage integrates into the bacterial genome, forming a structure called a prophage. **(B)** Scheme of site-specific recombination mediated by the phage in the bacterial host. The integrase performs precise recombination between an *attB* (bacterial attachment site) and an *attP* (phage attachment site). The result is the integration of the phage into the host genome and the formation of hybrid sequences *attL* (attachment Left site) and *attR* (attachment Right site).

Viruses are the most numerous microorganisms and phages are the reservoir of most of the genetic diversity in the sea, with about 10^30^ particles present in the oceans alone (Suttle, 2007). In addition, they can infect a wide variety of prokaryotes and can be found in soil, ocean, and extreme environments (Chibani-Chennoufi et al., 2004; Mills et al., 2013). Phages have great biotechnological relevance due to the multifunctionality of their enzymes and environmental relevance by altering cellular metabolism and influencing the cycling of chemical elements such as nitrogen, oxygen, and carbon (Warwick-Dugdale et al., 2019; Wang et al., 2022; Bisen et al., 2024). Moreover, these viruses have different characteristics in size, morphology, and genomic organization (Hatfull and Hendrix, 2011; Simmonds and Aiewsakun, 2018; Zhu et al., 2024). Phages are classified according to their morphological characteristics, their genetic material content, the location where they can be found and the bacterial species they can infect (Hatfull and Hendrix, 2011). They all comprise a DNA genome encased in a shell of phage-encoded capsid proteins, which protect the genetic material and mediate its delivery to the next host cell (Grigson et al., 2023; Turner et al., 2024). With the advancement of technology, electron microscopy allowed the detailed visualization of several types of phages, some of which appear to have “heads”, “legs” and “tails” (Figure 1A). Despite this appearance, phages are immobile and rely on Brownian motion or pedesis to reach their target host (Kasman and Porter, 2022). As a result of billions of years of coevolution between phages and their prokaryotic hosts, various mechanisms of attack and defense have been developed by both (Bernheim and Sorek, 2018). In nature, this coevolution leads to a constant “conflict” between the two groups, with the bacteriophages trying to find ways to infect the bacteria and the bacteria trying to defend themselves against the phages (Piel et al., 2022; Butt et al., 2024; Patel et al., 2024; Siedentop et al., 2024), although their dynamic interactions are still not fully understood (Sabino et al., 2020). This can result in a wide variety of bacteriophages and bacteria, each adapted to a particular environment or host. Phages are identified into two groups: lytic, or virulent, and lysogenic, or temperate, phages (Baaziz et al., 2024). Its biological cycle involves bacterial adhesion and invasion. As an obligatory intracellular parasite of a bacterial cell, phages can replicate through the biological cycles in the prokaryotic cell: lytic and lysogenic (Figure 1). The bacteriophage lytic cycle significantly affects bacterial mortality and nutrient cycling, as at the end of the process virions are released that can infect other bacterial cells and the host cell is destroyed. However, the size of the virion burst can vary significantly depending on the characteristics of the phage, the bacterium against which the bacteriophage is directed, and the environments in which the bacteriophage-host relationship occurs (Weinbauer, 2004). Lysogenic activity, on the other hand, is characterized by the integration of the viral genetic material (prophage) into the host chromosome and, when the cell divides, the transmission of the viral genetic material to the daughter cells and the host can lead to the distribution of beneficial characteristics for their hosts, such as resistance to antibiotics and phages or increased virulence (Bailey et al., 2024; Rostøl et al., 2024). However, under certain conditions, temperate phages can either multiply via the lytic cycle or remain dormant in the host cell (Young, 2013; Fogg et al., 2014; Doore and Fane, 2016). Lysogenic activity is less common than the lytic cycle, but it can be important in some cases, such as in the spread of genes between bacteria. The phage genome integration mechanism is mediated by phage-encoded integrases which, with no need of any other phage-encoded factors, catalyze highly site-specific, unidirectional recombination reactions (Figure 1B). Both integration and excision require Large Serine Recombinase (LSRs), the enzyme that mediates site-specific DNA recombination. LSRs are characterized as bacteriophage integrases responsible for inserting viral DNA into the bacterial chromosome and excising host DNA (Groth et al., 2004; Smith, 2015). This review is designed to deliver a comprehensive exploration of Large Serine Integrases (Ints), encompassing their origins, discovery, functionality, structure, and applications. The fundamental objective is to amplify the existing repertoire of proteins within this class capable of regulating gene expression in eukaryotic cells. Lastly, by providing a robust and inclusive analysis, this review will offer vital insights, engender further research, and potentially pave the way for novel practical applications of LSRs in the realm of gene expression control.

## 2 Recombinases: unraveling their functional spectrum

Unlike homologous recombinases that facilitate recombination between similar sequences, site-specific recombinases (SSRs) are a group of enzymes specialized in promoting recombination exclusively between specific recognition sites. SSRs encompass a series of recombination processes involving mutual exchanges between defined sites in the DNA sequence (Grindley et al., 2006; Wang et al., 2011). These recombinases are present in various hosts and play crucial regulatory roles. Among the most commonly used are those found in phages, such as Cre, λ integrase, phiC31 integrase, Bxb1 integrase, and Flippase recombinase (FLP) in the yeast *Saccharomyces cerevisiae*. SSR’s (Site-specific recombinase systems) have been discovered in bacteria and yeast to promote a number of functions, including the phase shift of certain bacterial virulence factors and the integration of bacteriophages into the host genome (Wang et al., 2011). The recombinase superfamily can be divided into two fundamental groups, based on the active amino acid within the catalytic domain of the enzymes in each family: the tyrosine (Tyr) and the serine (Ser) recombinases (Gaj et al., 2014). Families can be further subdivided into members based on size or the mechanisms used (Wang et al., 2011). The family of tyrosine recombinases are the most widespread among prokaryotes (Bacteria and Archaea), and are also found in eukaryotes, sharing a catalytic domain with an easily recognized motif. Tyrosine recombinases can be bidirectional or unidirectional. Bidirectional recombination occurs between two identical sites, while unidirectional ones act at different *attB* and *attP* sites, resulting in an irreversible recombination. This reaction can be reversed in the presence of accessory (helper) proteins known as excisionases (Wang et al., 2011). The excision mechanism of this type of integrase is based on the formation of an intermediate state of the four DNA strands known as *Holliday junction*, similar to what occurs in homologous recombination (Grindley et al., 2006). The most studied phage tyrosine recombinases are the integrases present in lambda phage, Cre-lox, R-RS, HK101, pSAM2 and FLP-FRT systems (Wang et al., 2011). The serine family integrases that perform unidirectional recombination need accessory proteins present on the phage to reverse the reaction. This integrase group binds to the phage DNA strand at the *attP* site and bacterial DNA at the *attB* site and thus promotes double-strand breakage concomitantly. In this way the viral DNA is integrated into the bacterial genome with the formation at the left end of the *attL* site and the *attR* site at the right end. Several serine-type integrases have been described, such as: phiC31, Bxb1, phiBT1, TP901, R4, MR11, A118, phiK38, Wβ e SPBC (Brown et al., 2011).

### 2.1 Serine integrases: mechanisms, structure, and their applications in biotechnology

**FIGURE 2:**
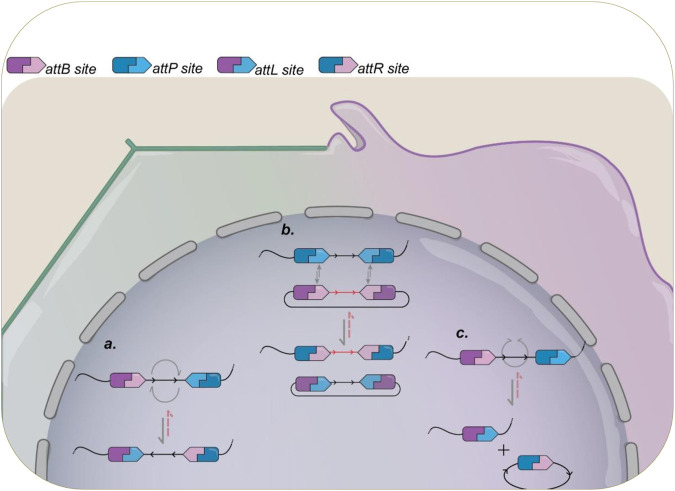
Schematic illustration of recombination activity promoted by serine integrases and the different possible outcomes. Already used in a variety of organisms, the outcome of a serine-integrase rearrangement can be controlled by the synthetic design of their *attB/P* sites. **(A)** when both sites are present in the same molecule, but with opposing orientation, their recombination will lead to an 180° inversion of the DNA sequence flanked by them. **(B)** Recombinase Mediated Cassette Exchange (RMCE) can be achieved when duplicates of each *att* site is present in different molecules. Upon recombination, the DNA sequences flanked will be swapped between the two constructions. **(C)** Different from DNA inversion, when both *att* sites are present in the same molecule, but presenting the same orientation, the recombination will result in excision of the target sequence, with formation of *attL* in the original molecule plus a circular DNA containing the excised DNA and the *attR* site formed. In all instances, the reverse reaction (red dotted lines) is only possible in the presence of cognate RDF.

Integration and excision reactions are remarkable because of their simplicity and high level of directionality. Serine integrases recombine substrates containing a phage attachment site (*attP*, attachment Phage or phage binding site) and a bacterial site (*attB*, attachment Bacteria or bacterium attachment site), with each integrase being able to act only on their specific recombination site pairs. Integrase recombination of *att* sites results in two hybrid sites, *attL* (attachment Left or left binding site) and *attR* (attachment Right or right binding site), each consisting of half *attP* sequence and half *attB* sequence. Depending on the orientation and location of the recombination sites within the same DNA molecule or in separate molecules, whether they are linear or circular, it is possible to perform various reactions. These reactions include integration, which involves the insertion of a DNA sequence (cassette) into a specific site within a DNA molecule; deletion, which involves the excision of a DNA sequence from a molecule; inversion, which refers to the inversion of a specific DNA sequence, altering its orientation; and RMCE (Recombinase-Mediated Cassette Exchange), which allows the replacement of a previously inserted DNA cassette with a new sequence from a circular DNA containing the gene of interest flanked by two copies of the same att site, allowing integration of fragments while still preserving the integrity of the remaining genome sequence. A diagram showing the recombination steps and outcomes for different designs is presented in Figure 2.

Although initially unidirectional, the presence of an additional protein encoded by the phage, called recombination directionality factor (RDF) can reverse the reaction by activating *attL* x *attR* recombination and inhibiting *attP* x *attB* recombination (Olorunniji et al., 2016; Olorunniji et al., 2017; Olorunniji et al., 2019). The identification of RDF proteins has proven to be a complex challenge. Studies have used artificial intelligence to analyze the degree of orthogonality in integrative and excisive responses of serine integrases, such as ϕC31, ϕBT1, and TG1, along with their respective RDFs. The results highlighted TG1 as the most active integrase with greater directionality (MacDonald et al., 2024). Among the characterized proteins, there are few homologous sequences, size variation, and a wide diversity in gene loci (Fogg et al., 2017). Some of the serine integrases and their respective known RDFs are phiC31 – gp3 (Fogg et al., 2018); Bxb1 – gp47 (Ghosh et al., 2006); TP901-1 – orf7 (Breüner et al., 1999); phiRv1 – Rv1584c (Bibb and Hatfull, 2002); SPBc – SprB (Abe et al., 2014); phiJoe – gp52 (Fogg et al., 2017); A118 – gp44 (Mandali et al., 2017); phiBT1 – gp3BT1 (Zhang et al., 2013). Moreover, a variety of Ints were identified, allowing the use of several of them concomitantly, and in this way, allowing the construction of genetic circuits (Bonnet et al., 2013). In this article, the authors report the construction of six logic gates (Boolean gates) in *Escherichia coli* using serine integrases Bxb1 and TP901 to control the orientation of terminators and promoters and, thus, allow or block the expression of the *gfp* reporter gene. A robust database search for other integrases was performed by Yang et al. (2014), finding 34 new ones and their cognate *attP* and *attB* sites, of which 11 were functional in *E. coli* DH10b, with orthogonal property, that is, one integrase does not recognize the site of another (Yang et al., 2014). Furthermore, they demonstrated that several integrases can be used sequentially in the same DNA segment to change the state of a transcriptional logic gate (Yang et al., 2014). A software for the design of genetic circuits, named Cello, was developed by Nielsen et al. (2016), which made it possible to test 60 serine integrases in *E. coli*, of which 45 worked as expected (Nielsen et al., 2016). However, most works are focused on prokaryotic cells. Although the natural environment of serine integrases is prokaryotic, these enzymes have been shown to be active in a variety of eukaryotic organisms (Gomide et al., 2020).

### 2.2 Tridimensional structure insights

**FIGURE 3:**
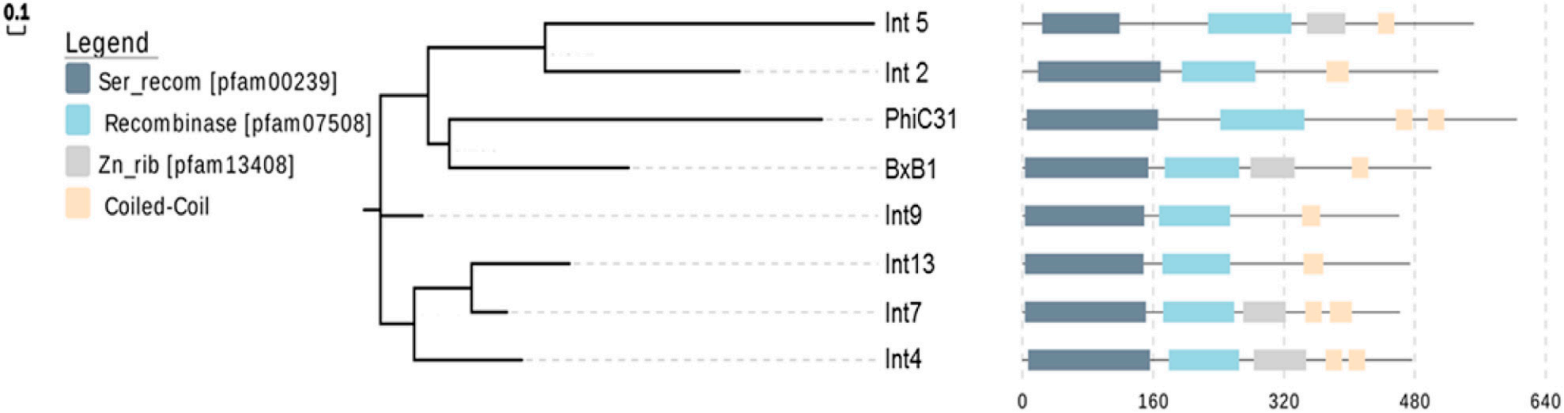
The phylogenetic tree and schematic representation of the domain architecture of the serine integrases. Phylogenetic tree of prophage integrases inferred using the Serine recombinase domain amino acid sequence and schematic representation of the conserved domains of the full-length integrases. Each domain is represented on scale. Ser_recom represents the serine recombinase domain and Zn_rib represents the recombinase zinc beta ribbon domain.

**FIGURE 4:**
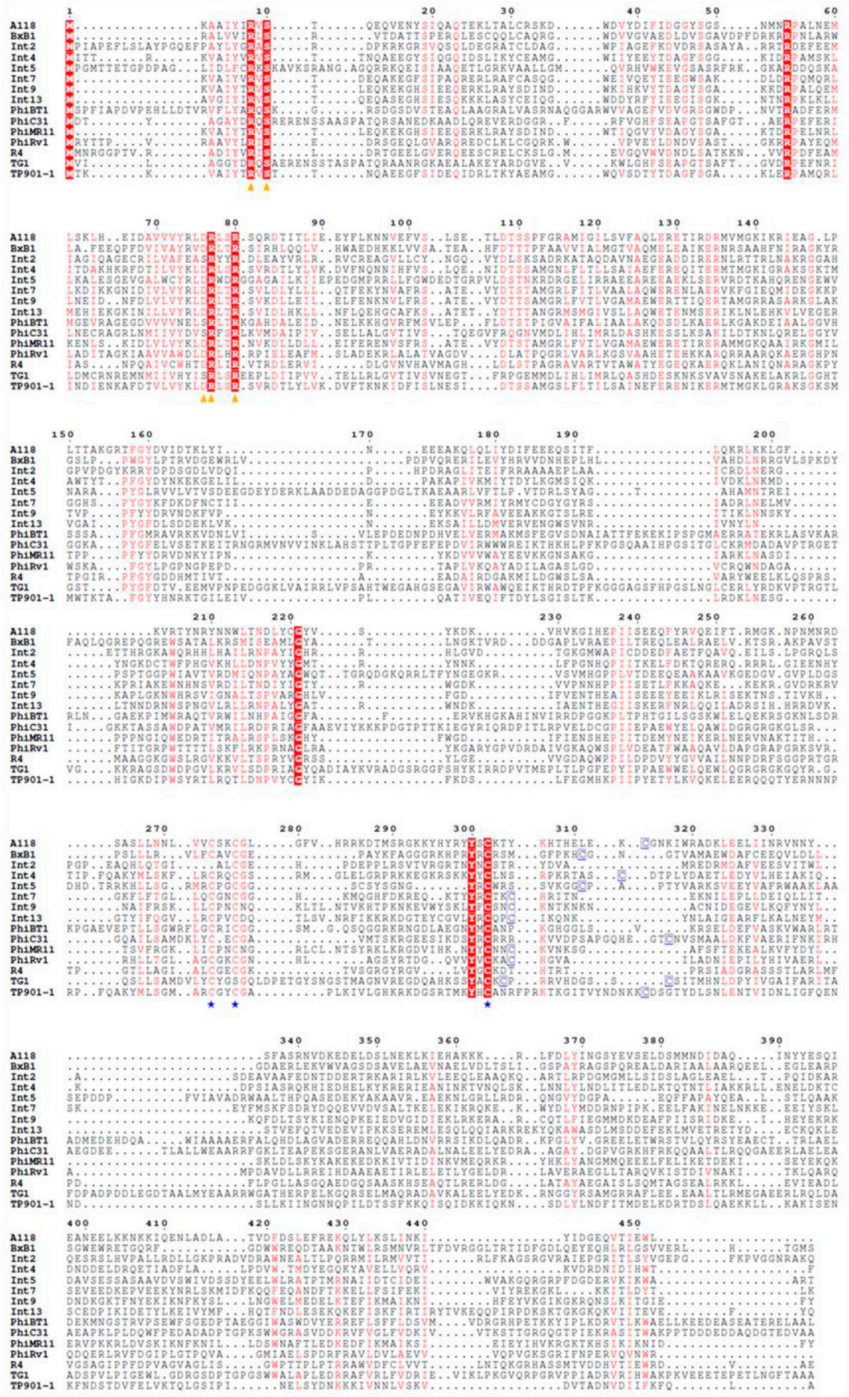
Amino acid sequence alignment of distinct Serine Integrases. Important catalytic domain residues are indicated with yellow triangles and cysteine residues are indicated with blue stars/squares. Conserved residues are highlighted in red.

**FIGURE 5:**
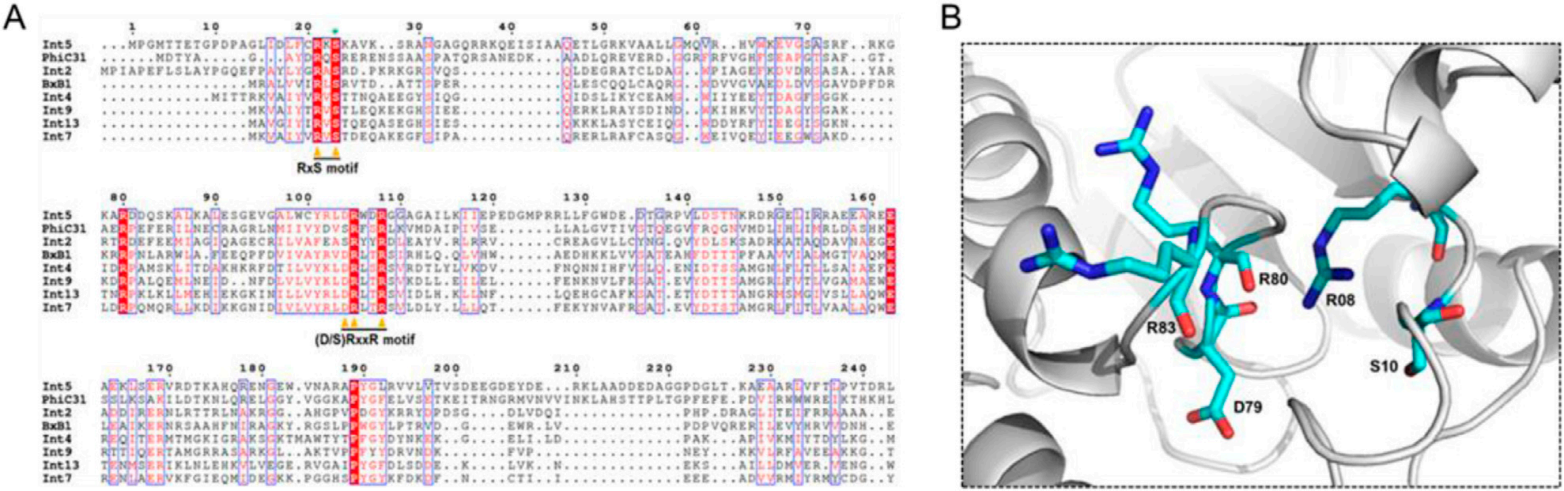
Representation of the three-dimensional structure of the N-terminal catalytic domain of Integrase 13 (Int13). **(A)** Amino acid sequence alignment of the N-terminal portion of different Serine Integrases. Important catalytic domain residues are indicated with triangles. Serine nucleophile (S10) is indicated with the green asterisk. Conserved residues are highlighted in red. **(B)** Structural homology modeling of the catalytic motif architecture: RxS… (D/S) RxxR of Int13.

The large serine recombinase family includes a diverse range of enzymes (Grindley et al., 2006; Smith, 2015). There are over 500 types of serine-integrase structures identified so far, and many domains remain unknown (DUFF domain) (Wang et al., 2023). However, the serine integrases commonly used in biotechnology have three well-known domains: Ser_recom [pfam 00239]; Recombinase [pfam 07508] and Zn-ribbon [pfam13408] (Figure 3). Furthermore, note that only some integrases have the Zn-ribbon domain (Figure 3). This is due to the presence or absence of four zinc-binding cysteine residues (blue stars), but all integrases are functional (Figure 4) (McEwan et al., 2011). The structural and biochemical features of these recombinases have been studied in detail to understand the mechanisms of their action and to develop novel tools for genetic engineering (Keravala et al., 2006; Bonnet et al., 2012; Van Duyne and Rutherford, 2013; Fogg et al., 2014; Rutherford and Van Duyne, 2014; Fan et al., 2016; Fogg et al., 2017; Stark, 2017). The tridimensional protein structures of a few serine integrases have been determined using X-ray crystallography and electron microscopy techniques (Ghosh et al., 2006; Rutherford et al., 2013; Gupta et al., 2017; Li et al., 2018; Mandali and Johnson, 2021). These structures reveal that the integrases adopt a homodimeric conformation with a conserved catalytic domain that contains the active site serine residue. The catalytic domain is connected to a DNA-binding domain, which recognizes the specific recombination sites in the DNA molecule. The enzyme is composed of two domains: N-terminal catalytic domain (NTD) and a C-terminal DNA binding domain (CTD) (McEwan et al., 2011). The catalytic domain contains a conserved serine residue that is essential for the recombination reaction, while the DNA binding domains recognize specific DNA sequences for recombination (*att* sites) (McEwan et al., 2009). In Figure 5A it is possible to observe the presence of conserved residues (yellow triangles): the N-terminal catalytic motif RxS … (S/D) RxxR. Moreover, these integrases have a flexible hinge region that allows for the formation of different conformations during the catalytic cycle (Figure 5B). This flexibility enables the integrase to adopt different configurations for the recognition and binding of DNA substrates, leading to the formation of different recombination products. Thus, serine integrases recognize and binds both *attB* and *attP* sites, then catalyzes a reaction that results in the integration, inversion or excision of the adjacent DNA. Further studies on the structure and function of these integrases will help in the development of new technologies for genetic manipulation and gene therapy.

## 3 Promising applications of serine integrases in eukaryotic systems

Among serine recombinases, serine integrases are considered a particularly fascinating subfamily. Pioneering studies with these enzymes were conducted to modify the genomes of *Streptomyces* bacteria at specific sites known as *attB*. Using serine integrase, researchers were able to stably integrate the pSAM2 plasmid into the bacterial chromosome at a predetermined location. This successful integration enabled the transmission of the integrated plasmid to descendant cells, expanding the possibilities for the application of these biotechnological tools (Merrick et al., 2018; Abioye et al., 2023). Over the course of more than 30 years, a wide variety of applications utilizing serine integrases in various eukaryotic organisms, including human cells can be found in the literature (Fogg et al., 2014; Snoeck et al., 2019). The use of these proteins has been applied in various ways, such as the removal of transgenes or the insertion of desired sequences at specific sites, referred to in these cases as “anchoring sites” or “landing platforms.” Additionally, these proteins are employed in cassette exchange mechanisms, DNA assembly, and in the activation or deactivation of gene expression through sequence inversion, enabling the construction of genetic switches (Groth et al., 2000; Stark, 2017). The main serine-integrases used for DNA editing in various eukaryotic organisms and the rearrangement mechanism applied are presented in Table 1.

**TABLE 1:** The main serine-integrases used for DNA editing in distinct eukaryotic cells.

Int	Mechanism	Organism	Delivery	Ref
68 new ones (Sh25; Si74; Bm99; Me99; Ma37; Nm60; Cc91; Vh19; Cs56; Bt24; No67; Fm04; Bu30; Ma05; Rh64; Cb16; uCb4; Ec03; Ec04; Ec05; Ec06; Ec07; Ef01; Ef02; Kp01; Kp03; Kp04; Kp05; Pa01; Pa03; Sa01; Sa02; Pf13; Td08; Se37; Ct03; Cd31; Ps40; Sa10; Td01; Enc3; Fp10; Ph43; Sm18; Cd16; Pf80; Bs46; Pf48; Rb27; Sa51; Bc30; Cd04; Cd15; Sa34; Pp20; Rl09; Efs2; Pf15; Ps45; Sp56; Dn29; Vh73; Em12; Pc64; Vp82; Cp36; Pc01; Enc9)	Integration	*Homo sapiens*	Transfection	Durrant et al. (2023)
A118	RMCE	*Saccharomyces cerevisiae*	Transformation	Xu and Brown (2016)
	RMCE	*Homo sapiens; Mus musculus*	Transfection; electroporation	Xu et al. (2013)
	Integration; excision	*Homo sapiens*	Transfection	Keravala et al. (2006)
BxB1	Integration	*Homo sapiens*	Transfection; electroporation	Yarnall et al. (2023)
	Integration	*Plasmodium falciparum*	Transfection	Adjalley et al. (2010)
	Inversion; excision	*Mus musculus; Drosophila melanogaster*	Transfection	Chow et al. (2021)
	Excision	*Homo sapiens*	Transfection	Chao et al. (2021)
	Inversion	*Homo sapiens; Bos indicus; Arabidopsis thaliana*	Transfection; transformation	Gomide et al. (2020)
	Integration	*Homo sapiens*	Transfection	Durrant et al. (2023)
	Integration	*Homo sapiens*	Transfection	Blanch-Asensio et al. (2022)
	Inversion	*Arabidopsis thaliana; Nicotiana benthamiana*	Agroinfiltration	Guiziou et al. (2023)
	Excision	*Nicotiana benthamiana*	Agroinfiltration	Oliveira et al. (2024)
	RMCE	*Saccharomyces cerevisiae*	Transformation	Xu and Brown (2016)
	RMCE	*Homo sapiens; Mus musculus*	Transfection; electroporation	Xu et al. (2013)
	Integration	*Mus musculus*	Nucleofection^a^	Zhao et al. (2014)
	Integration; excision	*Homo sapiens*	Transfection	Keravala et al. (2006)
	Inversion; excision; integration	*Schizosaccharomyces pombe*	Electroporation	Thomson and Ow (2006)
	Integration	*Plasmodium falciparum*	Transfection	Nkrumah et al. (2006)
	Integration	*Plasmodium falciparum*	Transfection	Spalding et al. (2010)
	Integration	*Oryza sativa; Nicotiana tabacum*	Biolistic	Li et al. (2016)
	RMCE	*Homo sapiens*	Nucleofection^a^	Farruggio et al. (2017)
	Inversion	*Homo sapiens*	transfection	Weinberg et al. (2017)
	RMCE	*Homo sapiens; Mus musculus*	Transfection	Geisinger and Calos (2015)
	Integration	*Cricetulus griseus*	transfection	Huhtinen et al. (2024)
	Inversion	*Arabidopsis thaliana*	Agrobacteria floral dip	Maranas et al. (2024)
	Integration	*Homo sapiens*	transfection	Hew et al. (2024)
EFC-1	Integration	*Homo sapiens*	Transfection	Yoon et al. (2016)
Int13	Inversion	*Homo sapiens; Bos indicus; Arabidopsis thaliana*	Transfection; transformation	Gomide et al. (2020)
	Inversion	*Nicotiana benthamiana*	Agroinfiltration	Oliveira et al. (2024)
Int2	Excision	*Homo sapiens*	Transfection	Chao et al. (2021)
	Inversion	*Homo sapiens; Bos indicus; Arabidopsis thaliana*	Transfection; transformation	Gomide et al. (2020)
Int4	Excision	*Homo sapiens*	Transfection	Chao et al. (2021)
	Inversion	*Homo sapiens; Bos indicus; Arabidopsis thaliana*	Transfection; transformation	Gomide et al. (2020)
Int5	Inversion	*Homo sapiens; Bos indicus; Arabidopsis thaliana*	Transfection; transformation	Gomide et al. (2020)
Int7	Excision	*Homo sapiens*	Transfection	Chao et al. (2021)
	Inversion	*Homo sapiens; Bos indicus; Arabidopsis thaliana*	Transfection; transformation	Gomide et al. (2020)
Int9	Inversion	*Homo sapiens; Bos indicus; Arabidopsis thaliana*	Transfection; transformation	Gomide et al. (2020)
	Inversion	*Nicotiana benthamiana*	Agroinfiltration	Oliveira et al. (2024)
MR11	RMCE	*Saccharomyces cerevisiae*	Transformation	Xu and Brown (2016)
	RMCE	*Homo sapiens; Mus musculus*	Transfection; electroporation	Xu et al. (2013)
phi370.1	RMCE	*Saccharomyces cerevisiae*	Transformation	Xu and Brown (2016)
	RMCE	*Homo sapiens; Mus musculus*	Transfection; electroporation	Xu et al. (2013)
phiBT1	Inversion; integration	*Homo sapiens*	Transfection	Farruggio and Calos (2014)
	RMCE	*Saccharomyces cerevisiae*	Transformation	Xu and Brown (2016)
	RMCE	*Homo sapiens; Mus musculus*	Transfection; electroporation	Xu et al. (2013)
phiC1	RMCE	*Saccharomyces cerevisiae*	Transformation	Xu and Brown (2016)
	RMCE	*Homo sapiens; Mus musculus*	Transfection; electroporation	Xu et al. (2013)
phiC31	Inversion; excision	*Homo sapiens; Mus musculus*	Transfection	Farruggio et al. (2012)
	Integration	*Ovis aries*	Transfection	Ni et al. (2012)
	Inversion; integration	*Homo sapiens*	Transfection	Farruggio and Calos (2014)
	Integration	*Cricetulus griseus*	Transfection	Ahmadi et al. (2016)
	Inversion	*Nicotiana benthamiana*	Agroinfiltration	Vazquez-Vilar et al. (2017)
	Excision	*Nicotiana benthamiana*	Agroinfiltration	Oliveira et al. (2024)
	Inversion	*Nicotiana benthamiana*	Agroinfiltration	Bernabé-Orts et al. (2020)
	Integration	*Bos taurus*	Transfection	Luo et al. (2015)
	Integration	*Sus scrofa*	Transfection	Bi et al. (2013)
	Integration	*Cricetulus griseus*	Transfection	Andreas et al. (2002)
	Inversion	*Homo sapiens*	Transfection	Weinberg et al. (2017)
	Integration	*Drosophila melanogaster*	Injection	Blanco-Redondo and Langenhan (2018)
	RMCE	*Saccharomyces cerevisiae*	Transformation	Snoeck et al. (2019)
	RMCE	*Homo sapiens*	Nucleofection^a^	Farruggio et al. (2017)
	Integration	*Homo sapiens*	Transfection	Durrant et al. (2023)
	Integration	*Homo sapiens*	Transfection	Blanch-Asensio et al. (2022)
	Inversion	*Arabidopsis thaliana; Nicotiana benthamiana*	Agroinfiltration	Guiziou et al. (2023)
	RMCE	*Saccharomyces cerevisiae*	Transformation	Xu and Brown (2016)
	Excision	*Danio rerio*	Injection	Lister (2010)
	Excision	*Danio rerio*	Injection	Lister (2011)
	RMCE	*Danio rerio*	Injection	Hu et al. (2011)
	Integration	*Mus musculus*	Nucleofection^a^	Zhao et al. (2014)
	RMCE	*Homo sapiens; Mus musculus*	Transfection; electroporation	Xu et al. (2013)
	RMCE	*Homo sapiens; Mus musculus*	Transfection	Geisinger and Calos (2015)
	RMCE	*Mus musculus*	Electroporation	Wei et al. (2011)
	RMCE	*Drosophila melanogaster*	Injection	Gohl et al. (2011)
	Integration	*Gallus gallus*	Electroporation	Dafhnis-Calas et al. (2005)
	Inversion; excision	*Gallus gallus*	Electroporation	Malla et al. (2005)
	Integration; excision	*Anopheles gambiae; Aedes aegypti; Drosophila melanogaster; Bombyx mori; Spodoptera frugiperda*	Transfection	Chompoosri et al. (2009)
	Integration	*Xenopus laevis*	Injection	Allen and Weeks (2005)
	Inversion	*Nicotiana benthamiana*	Agroinfiltration	Bernabé-Orts et al. (2020)
	Integration	*Arabidopsis thaliana*	Agrobacteria floral dip	De Paepe et al. (2013)
	Excision	*Homo sapiens*	Transfection	Chao et al. (2021)
	Inversion	*Homo sapiens; Bos indicus; Arabidopsis thaliana*	Transfection; transformation	Gomide et al. (2020)
	Inversion	*Arabidopsis thaliana*	Agrobacteria floral dip	Maranas et al. (2024)
	Inversion	*Homo sapiens*	Transfection	Hong et al. (2024)
	Integration	*Homo sapiens*	Transfection	Hew et al. (2024)
phiFC1	Integration; excision	*Homo sapiens*	Transfection	Keravala et al. (2006)
phiK38	RMCE	*Saccharomyces cerevisiae*	Transformation	Xu and Brown (2016)
	RMCE	*Homo sapiens; Mus musculus*	Transfection; electroporation	Xu et al. (2013)
phiRV	RMCE	*Saccharomyces cerevisiae*	Transformation	Xu and Brown (2016)
	RMCE	*Homo sapiens; Mus musculus*	Transfection; electroporation	Xu et al. (2013)
	Integration; excision	*Homo sapiens*	Transfection	Keravala et al. (2006)
R4	RMCE	*Saccharomyces cerevisiae*	Transformation	Xu and Brown (2016)
	RMCE	*Homo sapiens; Mus musculus*	Transfection; electroporation	Xu et al. (2013)
	Integration; excision	*Anopheles gambiae; Aedes aegypti; Drosophila melanogaster; Bombyx mori; Spodoptera frugiperda*	Transfection	Chompoosri et al. (2009)
SPBC	RMCE	*Saccharomyces cerevisiae*	Transformation	Xu and Brown (2016)
	RMCE	*Homo sapiens; Mus musculus*	Transfection; electroporation	Xu et al. (2013)
TG1	RMCE	*Saccharomyces cerevisiae*	Transformation	Xu and Brown (2016)
	RMCE	*Homo sapiens; Mus musculus*	Transfection; electroporation	Xu et al. (2013)
	Inversion; integration	*Homo sapiens*	Transfection	Farruggio and Calos (2014)
TP901	Excision	*Homo sapiens*	Transfection	Chao et al. (2021)
	Inversion	*Arabidopsis thaliana; Nicotiana benthamiana*	Agroinfiltration	Guiziou et al. (2023)
	RMCE	*Saccharomyces cerevisiae*	Transformation	Xu and Brown (2016)
	RMCE	*Homo sapiens; Mus musculus*	Transfection; electroporation	Xu et al. (2013)
	Inversion; excision; integration;	*Schizosaccharomyces pombe*	electroporation	Thomson and Ow (2006)
U153	Integration; excision	*Homo sapiens*	Transfection	Keravala et al. (2006)
	Inversion; excision; integration	*Schizosaccharomyces pombe*	Electroporation	Thomson and Ow (2006)
Wβ	RMCE	*Saccharomyces cerevisiae*	Transformation	Xu and Brown (2016)
	RMCE	*Homo sapiens; Mus musculus*	Transfection; electroporation	Xu et al. (2013)

^a^ Nucleofection allows the delivery of cargo DNA, directly inside the nucleus of the cell by electroporation.

### 3.1 Yeast

*Saccharomyces cerevisiae* is a widely studied model organism in biotechnology due to its ease of use, short life cycle, and expression capabilities. It can be used for the production of biofuels, metabolic engineering, and as a model for studying human diseases. phiC31, a type of serine integrase, has been utilized in the yeast *S. cerevisiae* to reintroduce specific markers, such as HIS5 and LEU2, through a technique called SIRE (*Serine Integrase Recombinational Engineering*). However, it has been observed that the efficiency of this system is restricted to low-copy vectors that carry the phiC31 integrase gene, likely because the enzyme exhibits toxic effects (Snoeck et al., 2019). In addition to the serine integrase phiC31 in yeast cells, the integrases ϕBT1, TP901, R4, Bxb1, MR11, A118, ϕK38, phiC31, Wβ, and SPBC demonstrated activity when expressed by *S. cerevisiae*. The authors conducted tests with fourteen serine integrases, some containing a nuclear localization signal (NLS), and observed that these enzymes were more efficient in promoting genomic integration reactions than simple homologous recombination. The group also noted that the presence of a nuclear localization signal (NLS) caused toxicity in the recombinases TG1 and BxB1. The most plausible explanation for these results was that the integrase in question was interacting with the nuclear genome in some way, becoming toxic. Finally, in the case of the recombinases Wβ and BL3, it was observed that the effect of the nuclear localization signal (NLS) was to reduce the toxicity of the integrase. This can be explained by the fact that these integrases do not rely on the NLS to enter the nucleus, and the presence of the NLS may compromise their ability to interact with the genome. This may occur due to a reduction in the integrase’s binding to ectopic recombination sites (Xu and Brown, 2016). Thomson and Ow (2006) selected three integrases from the extensive serine subfamily: Bxb1, TP901-1, and U153 (Thomson and Ow, 2006). This system has been shown capable of performing excision, inversion, and integration reactions in strains of *Schizosaccharomyces pombe*, such as FY527. In conclusion of the study, the functionality of these recombination systems in yeast could be applied, for instance, to invert DNA sequences and regulate gene expression. Given the potential of these systems in yeast, authors suggested they could be developed as tools for performing specific rearrangements in the genomes of plants and animals (Thomson and Ow, 2006).

### 3.2 Mammals

An important work with serine integrases in mammalian organisms involved experiments with fifteen integrases in human fibrosarcoma HT1080 cells and mouse embryonic stem cells. Initially, authors performed assays with *E. coli* in the presence of a reporter plasmid to confirm the activity of each integrase and its cognate binding sites by expressing each integrase gene in bacterial strains. The results confirmed that all the integrases and their binding sites were active in *E. coli* (phiC31, Bxb1, φBT1, φC1, MR11, TP901–1, R4, A118, φRV, TG1, φ370.1, Wβ, BL3, SPBc and K38). However, only four integrases (Bxb1, phiC31, R4, and φBT1) have demonstrated the ability to mediate precise integration at a specific site of the genomic DNA in HT1080 and ES cells. The results indicated that the Bxb1 integrase is the most efficient and precise, followed by phiC31, while the R4 and φBT1 integrases exhibited lower efficiency and precision (Xu et al., 2013). Weinberg et al. (2017), using different recombinase enzymes, designed 113 genetic circuits for eukaryotic cells, such as human embryonic kidney cells (HEK293T) and Jukart T cells, achieving 96.5% functionality. These data demonstrate the ability of serine integrates as biological tools for diverse applications, from genome engineering to the construction of biocomputers (Brown et al., 2011; Weinberg et al., 2017). Due to their directionality, recombination efficiency and simple DNA sequence requirements, they have been adopted for applications in the fields of molecular genetics, biotechnology and synthetic biology (Fogg et al., 2014). Yoon et al., (2016) characterized EFC-1 as a new serine integrase that can be used for site-specific recombination. Assays were conducted both *in vitro* and *in vivo* with HEK293 cells. PCR analysis and confocal microscopy confirmed the efficiency of integration in cells and determined the minimum lengths of the *attP* and *attB* sites. Recently, a study conducted by Gomide et al. (2020) investigated the use of six serine integrases utilizing EGFP fluorescence as a measure and molecular analysis of *attL*/*attR* site formation after integrase activation in HEK 293T cells. Additionally, as a clinically relevant proof of concept, evaluations of one-way gene switches using these integrases were also performed in peripheral blood mononuclear cells (PBMCs), neural stem cells (NSCs) differentiated from induced pluripotent stem cells, and undifferentiated human embryonic stem cells (hES, BR-1 cell line) (Gomide et al., 2020). The phiC31 integrase was also utilized for integration into endogenous “pseudosites”, regions with similar sequence of an target site that despite lacking perfect match to the correct site can still be rearranged by an integrase in specific cellular contexts. The researchers assessed the occurrence of homologous recombination mediated by the phiC31 integrase between *attB* and pseudo *attP* sites in sheep cells (Ni et al., 2012). In this study, a cellular therapy strategy was implemented using genetically corrected induced pluripotent stem cells (iPSCs). The research group explored a mouse model called mdx, which exhibits Duchenne muscular dystrophy, a recessive X-linked disease (Xp21.1 locus). To achieve this, they utilized phiC31 integrase to reprogram the genome at a safe site in mdx fibroblast cells, and the Bxb1 integrase to insert the complete therapeutic dystrophin cDNA into the iPSCs genome (Zhao et al., 2014). In the study conducted by Luo et al. (2015), the aim was to produce transgenic cattle with high expression of recombinant human serum albumin (HSA) in milk. This was achieved by using the phiC31 integrase system and somatic cell nuclear transfer (SCNT). During the study, bovine fetal fibroblast cells (BFFs) were co-transfected with the specific mammary expression plasmid pIACH(-), which contained the attB recognition site for the phiC31 integrase, along with the integrase expression plasmid pCMVInt (Luo et al., 2015). Six serine integrases, namely, Int2, Int4, Int5, Int7, Int9, and Int13, were evaluated by measuring EGFP fluorescence and analyzing the formation of *attL*/*attR* sites after integrase functionality in bovine primary fibroblasts. This study aimed to broaden the repertoire of proteins within this class that are capable of activating or deactivating genetic switches in mammalian cells (Gomide et al., 2020).

The detection of variability in integrase activity and its impact on the expression of downstream genes could have significant implications for the design of genetic circuits and for the use of these enzymes in gene therapy or other applications of Synthetic biology. Recently, a study established a characterization framework that includes an experimental protocol, mathematical models, and a computational pipeline for evaluating the main enzymatic parameters of serine integrases, using real-time measurements of fluorescent protein levels in HEK293 cells. The integrases evaluated were: int7, int2, int4, TP901, Bxb1, and phiC31 (Chao et al., 2021). The model identified three independent parameters: expression level, catalytic rate, and substrate affinity, which together explain a substantial amount of the differences observed in enzymatic activities. Among the results, the Bxb1 integrase showed a higher catalytic rate than phiC31, despite phiC31 being expressed at higher levels. The researchers propose that recombinases possess diverse characteristics that influence recombination efficiencies. As an example, int7, whose activity is virtually imperceptible, is expressed at a high level, indicating that its low final recombination efficiency is due to the protein’s functionality, not merely deficient expression. Conversely, phiC31, the recombinase with the highest final recombination efficiency, seemingly catalyzes recombination relatively slowly and relies on extremely high expression for its high efficiency, while int4 had a high catalytic rate but low expression. The team also tested expression vectors using a strong promoter (CMV) or a weak promoter (Ub) to enhance the expression of the integrases in cells. The researchers concluded that the expression level and binding affinity of serine integrases affect the expression of the downstream reporter gene, which could skew the results of the endpoint assay from directly reflecting the actual occurrence of DNA recombination (Chao et al., 2021).

Another study was the first to investigate the potential use of phiC31 to identify and utilize specific integration sites, called pseudo *attP* sites, in the porcine genome (Bi et al., 2013). The group conducted experiments in the PK15 cell line, transfecting plasmids containing phiC31 *attB* and *attP*, and obtained a maximum measured frequency of intramolecular recombination in the cells. They concluded that the selected integrase functioned safely to modify the cellular genome. Furthermore, through the Alamar Blue assay, they demonstrated that the integration of the transgene at these specific sites had little impact on cell proliferation, as no aberrant morphology or abnormal proliferation were observed in the transgenic cell lines. The group established an ideal model to study the effect of the position of an identical transgene in different chromosomal contexts. These findings also serve as a foundation for targeted porcine genomic engineering and can be used to produce genetically modified pigs for agricultural and biomedical purposes (Bi et al., 2013).

The Cre/loxP systems derived from bacteriophage and the FLP/FRT recombinase system derived from yeast have been utilized for introducing genetic modifications in various cell models. In 2002, Andreas S. et al. conducted experiments to compare the efficiency of the phiC31 with the Cre and FLPe recombinases in mammalian cell recombination. Chinese hamster ovary (CHO) cells were transiently transfected with the enzymes (phiC31, Cre, and FLPe) to evaluate their capability in catalyzing DNA recombination. Additionally, reporter cells were generated by stable transfection of NIH 3T3 cells to determine their efficiency in stable integrated targets (Andreas et al., 2002). The phiC31 integrase was modified by adding a nuclear localization signal (NLS) to investigate whether the fusion of NLS could further enhance the efficiency of the recombination process. Two different NLS fusions were created: one with an N-terminal NLS called (NLS-phiC31) and another version with a C-terminal NLS called (phiC31-NLS) to examine if the position of the NLS would influence recombinase activity. The research results indicated that the modified phiC31 integrase, with the addition of a C-terminal NLS, exhibited higher efficiency compared to the Cre and FLPe recombinases in mammalian cell recombination (Andreas et al., 2002).

Rheumatoid Arthritis (RA) is a widely recognized systemic autoimmune disease that is incurable. The etiology of RA remains uncertain, but it is commonly associated with genetic and environmental factors (Ding et al., 2023). Keravala et al. (2006) explored the use of the phiC31 integrase to mediate genetic integration in cultured synovial cells and to enhance genetic expression in rabbit joints. They utilized Hig82 cells, an adherent cell line derived from rabbit synovium, and primary human synovial cells obtained from RA patients for *in vitro* and *in vivo* experiments (Keravala et al., 2006). Donor plasmids that expressed the marker gene and contained the *attB* site, along with the plasmid expressing the phiC31 integrase, were injected, through the patellar tendon, into the hind joints of female rabbits. The results showed that the phiC31 integrase was effective in integrating into synovial cells and led to a significant increase in gene expression in the rabbits’ joints. This suggests that phiC31 integrase could be a promising strategy for gene therapy and the treatment of joint diseases (Keravala et al., 2006).

In this review article, we highlight the integrases Bxb1 and phiC31, regarded as the primary serine recombinases to the date. However, the reduced efficiency and the limited number of known serine integrases substantially restrict their utility as tools for DNA integration in mammalian genome engineering and other eukaryotic cells. Durrant, M.G. et al., (2023), carried out the systematic identification and characterization of a vast number of large serine recombinases (LSRs), derived from mobile genetic elements. In this study, a computational method was developed to identify thousands of new serine integrases and their attachment sites, expanding the known diversity of LSR by over 100 times and enabling the prediction of their site-specificities (Durrant et al., 2023). Tests in human cells, including K-562 lymphoblastoid cells and HEK293 cells, were conducted, classifying them according to genomic targeting. In total, 146,028 genomes from bacterial isolates available in the RefSeq database of the National Center for Biotechnology (NCBI) were analyzed. A series of new LSRs were identified, including Kp03 and Pa01, which outperform the previously characterized Bxb1 by 2–7 times in episomal and chromosomal integration efficiency. The LSR database may also include candidates capable of targeting non-human genomes, encompassing plants, eukaryotic microbes, insects, birds, among other model or non-model study organisms, which could facilitate stable transgenesis in a variety of organisms (Durrant et al., 2023). The group demonstrated that their findings are more extensive compared to previous techniques that relied solely on prophage annotations (Yang et al., 2014). The first study of hybridization with serine integrases to demonstrate chimeric activity of serine integrases addresses the engineering of chimeras to obtain activity in both *E. coli* cells and mammalian cells, specifically HeLa cells (Farruggio and Calos, 2014). Chimeras were created from three characterized members of the serine integrase family: phiC31, phiBT1, and TG1, by combining their amino and carboxy-terminal portions. The authors constructed several binary hybrids using arrangements that involve some portion of the phiC31 integrase and tested for activity in *E. coli* and/or HeLa cells with inversion reporter assays. The chimeras constructed were phiC31-phiBT1 (CB), phiBT1-phiC31 (BC), phiC31-TG1 (CT), and TG1-phiC31 (TC). Hybrids of three out of the four tested architectures—BC, CT, and TC chimeras—showed activity in *E. coli* at hybrid and/or parental *att* sites. The BC chimeras also demonstrated efficiency in HeLa cells, both in extra-chromosomal assays and at pseudo-sites. This study concluded on the degree of structural compatibility between the catalytic and C-terminal domains of phiC31, phiBT1, and TG1 integrases, and established foundations for using hybridization to create serine integrases with new specificities. Farruggio et al. (2012) were also the first to demonstrate the activity of the serine integrase on *attL* x *attR* sites in mammalian cells, including human cell lines (HeLa and HEK293) and mouse cells (NIH3T3). They validated phiC31 RDF as a new tool that would enable future studies to explore phiC31 integrase recombination in both direct and reverse directions (Farruggio et al., 2012). Farruggio et al. (2017) developed an RMCE protocol, called dual integrase cassette exchange (DICE), using phiC31 and Bxb1 integrases in human induced pluripotent stem cells (hiPSCs) to efficiently exchange specific genetic cassettes (H11 safe harbor site in the human genome) (Farruggio et al., 2017). The DICE system aims to allow specific and efficient exchange of genetic cassettes between different genomic locations. In addition, it has the ability to perform several genetic modifications in parallel, using different integrations and recombination sites. This facilitates the analysis of multiple genetic factors in a single experiment, increasing efficiency and reducing experimental variability (Farruggio et al., 2017; Zhu et al., 2014). The group also developed an RMCE protocol using phiC31 and Bxb1 integrases with the DICE method in other cell types, including the H9 female human embryonic stem cell line, gamma-irradiated CF1 mouse embryonic fibroblast feeder cells, and gamma-irradiated DR4 mouse embryonic fibroblast feeder cells (Geisinger and Calos, 2015). Overcoming the challenges of inserting multiple transgenes or genomic fragments of unlimited size into specific locations in mammalian cells, an innovative platform called STRAIGHT-IN (Serine and Tyrosine Recombinase Assisted Integration of Genes for High-Throughput Investigation) was developed to enable targeted genomic integration of large DNA payloads in human-induced pluripotent stem cells (hiPSCs). This methodology combines serine integrases (Bxb1 and phiC31) with CRISPR/Cas9-mediated homologous recombination to achieve precise and site-specific replacement of large genomic regions. The authors demonstrated that there is no restriction on the size of the DNA payload that can be integrated, overcoming one of the main limitations of other commonly used DNA integration systems, such as viral vectors and programmable nuclease knock-ins. Results showed the efficient integration of DNA sequences ranging from 14 to 50 kb in length. Thus, the STRAIGHT-IN platform can significantly simplify the generation of cell lines or animal models containing large and complex genetic circuits, as well as facilitate multiplex genetic assays (Blanch-Asensio et al., 2022). Wei et al. (2011) used the RMCE method to generate a strain of mice called p53 Platform. These mice contain specific *attP* sequences for the phiC31 integrase at the Trp53 locus. The Trp53 or TP53 gene encodes the p53 protein, which plays a crucial role in cell cycle regulation and cancer prevention. To produce somatic cell lines with endogenously controlled expression of mutant p53, cassette exchange mediated by the phiC31 integrase was performed in embryonic stem (ES) cell lines from mice. This approach allowed precise insertion of DNA sequences at the Trp53 locus, facilitating the investigation of the effects of different mutations in the p53 gene (Wei et al., 2011). Present programmable addition via site-specific targeting elements (PASTE) system combines the precise targeting of CRISPR-Cas9 with the Bxb1 integrase in primary human hepatocytes and T cells. This method was developed to eliminate a defective gene and replace it with a functional gene without inducing double-strand breaks in the DNA. The PASTE system achieved programmable integration of genetic payloads of up to approximately 36 kb in a single delivery reaction (Yarnall et al., 2023). Chow et al. (2021) presented an innovative system called integrase-editable memory by engineered mutagenesis with optical *in situ* readout (intMEMOIR) (Chow et al., 2021). This system uses the serine integrase Bxb1 to perform irreversible nucleotide edits and fluorescence *in situ* hybridization (FISH) to read these mutations. This design generates various digital and permanent edit states that can be stably inherited over many cell cycles. The synthetic barcode enabled the *in situ* reconstruction of lineage relationships in mouse embryonic stem cells (mES) and in fly neural development. The editing deletes or inverts its target region, encoding information in three-state memory elements: “not mutated,” “inverted,” or “deleted.” To create a “trit,” the authors flanked a barcode sequence with an inverted pair of *attP* sites at one end and an *attB* site at the other, allowing Bxb1-mediated recombination to produce irreversible deletion or inversion of the barcode (Chow et al., 2021). The ability to visualize cell lineage relationships, distribution, and interactions between cells in native tissue provides deep insight into cell fate determination during development, homeostasis, and disease.

### 3.3 Insects: dipterans and lepidopterans

Research in the field of genetic engineering has been dedicated to exploring techniques for insect control. The goal is to develop safe and efficient approaches to reduce disease transmission in animals and humans, protect public health, and combat agricultural pests. In a study involving the model organism *Drosophila melanogaster*, a genomic integration method using the integrase enzyme phiC31 was employed to direct transgenes to specific locations in the genome. Two transgenic strains of *D. melanogaster* were generated using the serine integrase phiC31, both in cultured cells (S2) and in embryos (Groth et al., 2004). In studies conducted by Chompoosr et al. (2009), it was found that the integrases phiC31 and R4 have the ability to facilitate site-specific directional recombination in various cell lines, such as *Aedes aegypti* (Aag2), *Anopheles gambiae* (Sua5B), *Drosophila melanogaster* (S2), the silkworm *Bombyx mori* (BmN4), and the fall armyworm *Spodoptera frugiperda* (Sf9 cells) (Chompoosri et al., 2009). An alternative approach to enable repeated and independent manipulation of two linked genetic loci in *Drosophila* was achieved through phiC31 integration. A second pair of attB/attP targeting and transgenesis vectors was introduced, operating in parallel and independently of existing tools. Two synthetic orthologous genes were genetically modified to include orthogonal *attB/attP* sites. The results demonstrated that the specificity, directionality, and efficiency of recombination of the introduced orthogonal *attB* ^CC^ /attP ^CC^ sites can be used in conjunction with the canonical *attB* ^TT^ /*attP* ^TT^ system for independent manipulation and analysis of two transgenesis targets simultaneously in the same organism (Blanco-Redondo and Langenhan, 2018). The InSITE (integrase swappable *in vivo* targeting element) system using RMCE with phiC31 integrase allowed the replacement of the GAL4/UAS binary expression system with analogous systems (such as hemi-driver split GAL4, GAL80, LexA, or QF) in fruit fly *D. melanogaster.* With the InSITE system, if a fly line with the desired expression pattern contains the wrong genetic element, the correct element can be swapped in with just a few simple crosses, without the need to inject any plasmids (Gohl et al., 2011).

### 3.4 Fish, amphibian and bird

Model organisms, such as *Danio rerio* and *Xenopus laevis*, have also been used in research with the phiC31 integrase. Ints were employed as a mediator for the recombination of transgenes containing *attP* and *attB* sites within cis-regulatory sequences, aiming to excise specific elements in zebrafish (Lister, 2010; Lister, 2011). RMCE was also evaluated in the model, using phiC31 integrase to efficiently mediate cassette exchange in both somatic and germline cells of zebrafish (Hu et al., 2011). Additionally, it was used to generate transgenic *Xenopus embryos* through co-injection of mRNA encoding the phiC31 integrase along with plasmid encoding *gfp* (Allen and Weeks, 2005). In the chicken DT40 cell line, the phiC31 integrase was employed to facilitate the efficient integration of plasmids and fragments up to 100 kb into vertebrate chromosomes. The results of this research demonstrated that the phiC31 integrase can be successfully used in the generation of transgenic chickens (Dafhnis-Calas et al., 2005). The nuclear import signal and the position within a prokaryotic recombinase can play a crucial role in its efficiency in mammalian cells. Previous studies with the phiC31 integrase in eukaryotic cells have also revealed a variable requirement for a nuclear localization signal (NLS). In chicken DT40 cells, this requirement is absolute, while in *D. melanogaster*, there appears to be no requirement at all (Groth et al., 2004; Dafhnis-Calas et al., 2005). The group also conducted experiments with chicken DT40 cells to design a minichromosome derived from the human Y chromosome, containing *attB* and *attP* sites flanking the centromeric alphoid DNA. They used the phiC31 integrase to promote the deletion or inversion of the centromeric interval. The results showed that the phiC31 integrase promotes efficient, irreversible, and site-specific long-range chromosomal rearrangement in vertebrate cells. Furthermore, they demonstrated that no pseudo-sites were detected in the chicken genome (Malla et al., 2005).

### 3.5 Plants

Li et al. (2016) developed a protocol for biolistic transformation for site-specific integration mediated by Bxb1 in tobacco (*Nicotiana tabacum*) and rice (*Oryza sativa*), demonstrating efficient gene insertions at these target sites (Li et al., 2016). To implement this system in rice, they generated lines through conventional transformation mediated by *Agrobacterium*, and various precise target sites in the rice genome were tracked. The GoldenBraid 3.0 (GB3.0) system was developed as a platform for assembling reusable genetic components for Plant Synthetic Biology, incorporating features of its synthetic parts (Sarrion-Perdigones et al., 2013). In this context, a GB3.0 database was established based on the GB2.0 assembly system. For the GB3.0 system, transient Luciferase/Renilla (Luc/Ren) agroinfiltration experiments were carried out in *N. benthamiana.* The group developed two movements to control transcription. The first one is based on the phiC31 integrase fused to activation (Gal4 or VP64) or repression phiC31 fused to an RD (BRD) domains (Vazquez-Vilar et al., 2017). In the second one, the phiC31 integrase and its counterpart RDF were used to switch between active or inactive states of reporter genes by inverting regulatory parts in *Nicotiana benthamiana* (Bernabé-Orts et al., 2020). This research marked the first reversible memory swap in entire plants based on the serine phiC31 integrase and its corresponding RDF. De Paepe et al. (2013) demonstrated that T-DNA integration occurred in the progeny of 9% of the T-DNA transformants obtained in *Arabidopsis* using the method called Iterative Site-Specific Integration (ISSI), which combines the activities of CRE recombinase and phiC31 integrase for efficient T-DNA integration (De Paepe et al., 2013). In another study, the functionality of six integrases: Int2, Int4, Int5, Int7, Int9, and Int13, as well as the phiC31 and Bxb1 integrases in a plasmid cotransfection system was proven to perform the 180° rotation of coding and promoter sequences of the designed genetic switches, thus controlling the expression of the *gfp* reporter in *Arabidopsis thaliana* protoplasts (Gomide et al., 2020). The gene expression control systems mentioned above in plants open a wide range of combinatorial possibilities for genetic circuits specifically designed for plants. Such systems allow for the monitoring of agricultural applications, as well as the activation of synthetic genetic networks that respond to biotic factors, such as diseases and pathogens, and abiotic factors. The development of lateral roots in plants serves as an excellent model for the application and study of gene expression, thanks to its well-defined transcriptional control mechanisms. In addition to their positioning, lateral roots exhibit structural characteristics linked to environmental adaptability, making them ideal candidates. To construct synthetic circuits in transgenic *Arabidopsis* lines, Guiziou et al. (2023) tested the efficiency of serine integrases phiC31, Bxb1 and Tp901. Their results indicated that phiC31 and Bxb1 integrations were constitutively expressed in all plant tissues and were orthogonal to each other (Guiziou et al., 2023). In contrast, the Tp901 integrase did not induce any changes in the targets that carried its recognition sequences, even with strong promoters and codon optimization. phiC31 integration was selected for further experiments in lateral root development. To facilitate this, the researchers developed an integrase toolbox, selecting promoters for several well-trained transcription factors expressed during the initial stages of lateral root initiation: ARF7, ARF19, LBD16 and GATA23. In addition, *N. benthamiana* was used for transient assays. The team also characterized two methods for adjusting the timing and level of integrase activity: the split-intein integrase and the estradiol-inducible integrase. These methods allow greater control over gene expression, increasing the potential for sophisticated synthetic biology applications in plants. Lastly, Oliveira et al. (2024) integrated a bimodular DNA excision and inversion circuit driven by four distinct serine integrases into the genome of *N. benthamiana*. The system, denoted Int-Plex@, demonstrated to be functional, allowing the precise excision and inversion of genomic DNA sequences. Through the application of BxB1, phiC31, Int9 and Int13, it was possible to activate specific modules to control the removal or inversion of DNA segments, showing the potential of the system as a tool for engineering genetic circuits and modulating metabolic pathways in plants in a controlled and reversible manner (Oliveira et al., 2024).

### 3.6 Protozoan

*Plasmodium falciparum* is the causative agent of malaria. The protozoan invades human erythrocytes, inducing a cascade of changes in these cells for its development and proliferation (Zheng et al., 2024). In this scenario, science has developed various tools for genetic manipulation of this organism. Nkrumah et al. (2006) developed *P. falciparum* strains with the *attB* site integrated into the cg6 gene, which encodes an important protein in the parasite’s life cycle, using the Bxb1 integrase (Nkrumah et al., 2006). The use of this integrase allowed for more efficient and precise integration compared to traditional genetic transformation methods, enabling the generation of transgenic parasites with controlled genetic insertions. Building on this work, Spalding et al. (2010) sought to validate the method by modifying Bxb1 integrase-mediated recombination in *P. falciparum*, using the H protein from the glycine cleavage complex (GCV) located in the mitochondria as an indicator (Spalding et al., 2010). They generated integrated cell lines expressing the H-GFP protein (HDd2 protein) and demonstrated the localization of this protein in the parasite’s mitochondria, thereby validating the efficacy of the recombination method. Balabaskaran-Nina and Desai (2018) described a new application strategy using the Bxb1 integrase to allow targeted gene substitutions through an intronic *attB* sequence within the gene of interest (Balabaskaran-Nina and Desai, 2018). The authors argue that this approach enables rapid specific site integration, encompassing the full spectrum of native genetic modifications and offering distinct advantages compared to other methods, including CRISPR-Cas9.

## 4 Discussion

There are several techniques used in gene editing research, such as the TALEN (Transcription activator-like effector nuclease), ZFN (Zinc Finger Nucleases), and the CRISPR/Cas9 systems. These methods use different proteins to direct restriction enzymes to cut DNA at specific locations. Each system has advantages and disadvantages, and the selection depends on the purpose of the genetic modification. However, it is still a difficult task to identify nucleases with high affinity and specificity. New methodologies are currently being used in Synthetic Biology to create new molecular approaches that lead to characterization, generating new components, cells, biomolecules, expression, and modification of genetic material (DNA/RNA). Genetic engineering offers promising applications in the areas of health, agriculture, and the environment, which requires the development of tools to improve the modulation of multiple genes. The use of serine integrases has been increasingly explored in Synthetic Biology studies because these enzymes can mediate specific recombination of DNA sequences, enabling the integration or excision of genes of interest at specific locations in the genome. A major advantage of using these recombinases is their high efficiency in inserting or removing DNA sequences, avoiding unwanted changes in the genome. This makes these enzymes a promising tool for various biotechnological applications, such as the production of transgenic organisms and gene therapies. It is essential to highlight that serine integrases of phage origin are valuable tools for promoting selective integration of genomic sequences into eukaryotic cells, combined with donor plasmids containing the *attB* recognition site with introduced genomic *attP* sites or pseudo-*attP* sites. Recent studies have shown that serine integrases can be successfully used in plants, human, and animal cells, paving the way for therapeutic and biotechnological applications. The applications may include various areas such as treatment of metabolic disorders, immune-mediated diseases, anticancer therapy, human or animal reproductive area, animal breeding and cloning in agriculture, among others. In addition, the requirement for recognition of two sites (*attB*/*attP*) for its action can be a minimizing factor for off-target gene editing. Despite the promising results observed in the use of these enzymes in eukaryotic cells, many questions remain to be investigated.
